# Incidence of tropical spastic parapesis/ HTLV-1 associated myelopathy/ (TSP/HAM) in patients followed in a Reference center in Salvador, Brasil

**DOI:** 10.1186/1742-4690-12-S1-P35

**Published:** 2015-08-28

**Authors:** Izabel Bou Teixeira, Ney Boa-Sorte, Ramon Kruschewsky, Thessika Araújo, Maria Fernanda Grassi, Bernardo Galvão–Castro

**Affiliations:** 1 Centro Integrativo e Interdisciplinar de HTLV (CHTLV), Escola Bahiana de Medicina e Saúde Pública; Salvador-Bahia-Brazil; 2 Laboratório Avançado de Saúde Pública, Centro de Pesquisas Gonçalo Moniz, Fundação Oswaldo Cruz, Salvador, Bahia, Brasil

## 

It is estimated that currently 5 to 10 million people are infected with HTLV-1 worldwide. In Brazil, Salvador is the city with the highest prevalence of HTLV-1 (2% in women and 1.2% in men). Tropical spastic paraparesis/ HTLV associated myelopathy (TSP/HAM) is an insidious and disabling chronic neurological disease. The incidence of TSP/HAM varies in different geographical regions with low rate in Japan and Caribbean islands and higher in Minas Gerais, Brazil. The incidence of TSP/HAM among patients attending the CHTLV in Salvador/BA, from 22 to 2013 was estimated. The outcome of interest was the evolution from asymptomatic status for TSP/HAM diagnosis, which was based on the Belem criteria. The follow-up for individuals that remained asymptomatic was the time between the year of the last medical consultation and the year of the HTLV-1 serological diagnosis and for those that developed TSP/HAM the time between the year of diagnosis of TSP/HAM and the year of HTLV-1 serological diagnosis. The exclusion criteria were a diagnosis of TSP/HAM at the first medical consultation, records with incomplete data and TSP/HAM possible diagnosis. From a sample of 243 patients, 91 were included. Two patients developed TSP/HAM definite and four had TSP/HAM probable diagnosis. The incidence density of TSP/HAM definite was 6.9 and 13 per 1,0 HTLV-1 infected individuals per year, respectively. The cumulative incidence to TSP/HAM definite was 2.29% while to TSP/HAM probable was 4.39%. The incidence rate found herein was higher than that observed in previous studies in Japan and Caribbean, however, similar to that reported in Brazil. More accurate cohort studies should be conducted to measure the actual risk of developing this disease.

